# Interactive Music Therapy on Stress Level Reduction in Women Submitted to IVF/ICSI. Prospective Randomized Study

**DOI:** 10.5935/1518-0557.20200068

**Published:** 2021

**Authors:** Eliamar Aparecida de Barros Fleury, Mário Silva Approbato, Maria Alves Barbosa

**Affiliations:** 1 Reproduction Human Laboratory. Obstetrics and Gynecology Dept.; Music and Scenic Arts School. Federal University of Goiás State, Goiânia, Brazil; 2 Reproduction Human Laboratory. Clinics Hospital. Obstetrics and Gynecology Dept. Faculty of Medicine. Federal University of Goiás State, Goiânia, Brazil; 3 Faculty of Nursing. Federal University of Goiás State, Goiânia, Brazil

**Keywords:** interactive music therapy, infertility, IVF/ICSI, stress

## Abstract

**Objective:**

To identify the effects of interactive music therapy on stress levels in women undergoing high complexity infertility treatments.

**Methods:**

Prospective randomized study involving 113 women treated in the Reproduction Human Laboratory of the Clinics Hospital of the Federal University of Goiás State, submitted to *in vitro* fertilization/intracytoplasmic sperm injection. We used Depression, Anxiety and Stress Scale, and Lipp’s Stress Symptoms Inventory for Adults. In the Intervention Group, we used small and easy to play percussive musical instruments, a guitar, voice, and a recorder. We used interactive music therapy approach individually, applied before baseline ultrasound scan, oocyte pick-up, and embryo transfer. We analyzed the data using the R. Paired *Student t-test* to compare the results.

**Results:**

Comparison of the stress levels by Depression, Anxiety and Stress Scale between the groups in the final moment of data retrieval resulted in 23.13 (SD±10.51; n=32) in the Control Group and 16.12 (SD±7.87; n=33) in the Intervention Group, being statistically different (*p*=0.004). Also in Lipp’s Stress Symptoms Inventory for Adults there was a significant stress reduction in 39% of the patients in the Intervention Group compared to a reduction of 14% in the patients of the Control Group (*p*=0.032). In this same measurement resulted that only 3% of the Intervention Group patients *versus* 23% of the Control Group patients (*p*=0.027) were in the exhaustion stage.

**Conclusion:**

Interactive music therapy was effective for stress reduction in women during assisted reproduction techniques.

## INTRODUCTION

Infertility is understood as a couple’s problem, awaking the interest in investigation about the damages caused by this disorder in man (Gollenberg *et al*., 2010), and the negative reflexes in the life quality of men, women, and couples (Chachamovich, 2009). Despite that, it is the woman who is generally more open to medical treatment for infertility and in some cases she continues to feel responsible for the diagnoses (Miranda, 2005; Peterson *et al*., 2014). It is also the women who suffer the collateral effects of medication (Finotti, 2011).

Different biological mechanisms related to or initiated by stress can cause reduction in fertility. However, it is difficult to establish cause-effect associations between this disease and stress, considering the multiplicity of causes of infertility, including male factors (Moreira *et al*., 2006; Moreira & Azevedo, 2010). With interest in the emotional issues of women, new therapies are close to this field, such as music therapy, which is a self-expressive, non-pharmacological therapy, with music as the main element of intervention (Fleury *et al*., 2015).

There are also studies in the field of health that use music through receptive methods, usually applied by a non-music therapist (Bradt *et al*., 2013). Both approaches - Music in Medicine and Music Therapy in Medicine - described by Dileo in 1999 (Bradt *et al*., 2013), are valuable for patient care, but are different in many aspects. Focused on therapeutic objectives, music therapy must be performed by an accredited professional in the field (graduate or postgraduate). The treatment is performed through a sound-musical bond (Ubam, 2018). The license in the field will allow the professional to articulate the fundaments of the specialty and technical knowledge to choose the specific methods that are more appropriate to the needs of the patient or target public that will receive the intervention (Fleury *et al*., 2016).

Music Therapy is a complementary therapy to medical practice in different specialties. However, after extensive literature search, we found no publications about *Interactive Music Therapy* in women undergoing assisted reproduction (Fleury *et al*., 2014), which justifies the present study. There is a large number of women who show stress, anxiety, and depression associated to infertility (both individual and couple infertility) (Peterson *et al*., 2014). This process deserves further investigation with specific approaches, such as interactive music therapy. The hypothesis of this study is that Interactive Music Therapy reduces the stress level in infertile women undergoing high complexity techniques. This is the first Brazilian study with this approach to be conducted with this population (Ferreira, 2018)[1].

## MATERIALS AND METHODS

This was a prospective randomized quantitative study, conducted with women undergoing *in vitro* fertilization/in tracytoplasmic sperm injection (IVF/ICSI) treatment, seen in the Reproduction Human Laboratory/Clinics Hospital. The Ethics Committee of the institution where it was carried out approved the project, and the participants signed the Free Informed Consent Form. Between January 2015 and May 2016, 208 patients were treated with probable indication for IVF/ICSI procedures. Out of this number, 113 women met the study’s inclusion criteria, being randomly distributed between Control Group (CG) and Intervention Group (IG).

The approach applied was Interactive Music Therapy (Barcellos, 1984), in 50-minute individual sessions, conducted before baseline ultrasound, oocyte pick-up, and embryo transfer. We used Musical Exploration (Barcellos, 2004), Non-referential Musical Improvisation (Bruscia, 2000), and Assisted Musical Composition (Barcellos, 2015). The instrumental resources used in the first intervention in IG were small, easy to play musical percussion instruments. In the second and third interventions, we used a classic acoustic Di Giorgio 18 guitar; a digital processing TG-85 Chromatic Tuner; a Zoom H1 Handy Portable Digital Recorder; voice as musical instrument (participant and researcher); sheets of paper and pen.

The therapeutic music interventions were recorded (data collection), according to previous authorization expressed by the participants in a Free Informed Consent Forms. Afterwards, the audios were transcribed, and the verbal content of the compositions were registered in writing. For data collection, we used the *Depression, Anxiety and Stress Scale* (DASS 21) and the Lipp’s Stress Symptoms Inventory for Adults (LSSI).

Before the IVF/ICSI, there is the follicular growth (follicle maturation). Then there is the oocyte aspiration for posterior fertilization. This process usually takes up to 14 days, considering until the pickup. Then, there is the fertilization in lab with embryo transfer. Therefore, all the process occurs in a period smaller than 30 days, the interval required for a second LSSI data collection. This justifies the procedure of utilizing this instrument only in the end of the retrieval.

## RESULTS

Table 1 shows the results associated to psychological stress. We ran student t-tests for DASS 21 variables.

**Table 1:** Distribution of rate and ranks of answer variables referring to auto perceived stress. Human Reproduction Laboratory HC/GO, 2015-2016.

Variable	Control	Intervention	*p*-value
DASS Stress*(Pre)	20.48±11.28 (n=50)	20.4±9.91 (n=50)	0.970
DASS Stress*(Post)	23.13±10.51 (n=32)	16.12±7.87 (n=33)	0.004

Values expressed in means ± standard deviation;

* Student t test.

Table 2 shows the intragroup analysis (paired sample) of IG patients. We have the mean, standard deviation, and *p*-value of auto perceived stress by the patients of the study.

**Table 2:** Distribution of answer variables referring to auto perceived stress, measured before and after music therapeutic intervention, intragroup, IG. Human Reproduction Laboratory HC/GO, 2015-2016.

	Intervention Group		
	Before	After	*p*-value
DASS STRESS*	19.58±9.91	16.12±7.87	0.012 (n=33)

Values expressed in means ± standard deviation;

* Student t-test.

We ran the same analysis with CG patients at the beginning and at the end of the retrieval period. Table 3 depicts this data.

**Table 3:** Distribution of answer referring to auto perceived stress, measured before and after the intervention phase, intragroup, CG. Human Reproduction Laboratory HC/GO, 2015-2016.

	Control Group		
	Before	After	*p*-value
DASS STRESS*	22.63±11.28	23.13±10.51	0.807 (n=32)

Values expressed in means ± standard deviation;

* Student t test.

LSSI showed that there was a significant stress reduction in 39% of the patients in the Intervention Group, compared to a reduction of 14% in the Control Group patients (*p*=0.032). In this same measurement method, only 3% of the Intervention Group patients *versus* 23% of the Control Group patients (*p*=0.027) were in the exhaustion stage.

## DISCUSSION

The present study was random prospective with a sample of 113 patients undergoing IVF/ICSI fertility treatment. It is a pioneer study, based in the Music Therapy in Medicine, in an interactive approach. The specific techniques used were Music Exploration, Non-Referential Instrumental Improvisation, and Assisted Music Composition. We did not find publications about interactive music therapy applied to women undergoing fertility treatment via IVF/ICSI. We found a handful of studies that used recorded music, with or without earphones, conducted with women in IVF/ICSI treatment. However, even though such therapies are called by the authors as Music Therapy interventions, after checking the methodology of these studies, they were found to be Music in Medicine, and not Music Therapy in Medicine (Bradt *et al*., 2013). The two approaches are different in several aspects, but are widely used by different medical specialties.

In general, Music in Medicine uses music by receptive methods (Bradt *et al*., 2013). It can be live music or recorded, with previously selected songs (Taets & Barcellos, 2010). In case of recorded music, the therapy can be with or without earphones. In Music Therapy in Medicine (Bradt et al., 2013), in an interactive approach (Barcellos, 1984), the subjects participate actively of the musical creation process during the intervention, and the bond occurs by musical-sounds.

Our results show the statistically meaningful difference between CG and IG at the end of the study (DASS; 0.004) (Table 1), stating the positive effect of interactive music therapy on the patients’ stress. The analyses in each group (intragroup) (Table 2) detected statistically meaningful differences in the stress level of IG patients (*p*=0.012; n=33). In this same method of analysis, conducted in CG (intragroup), the results showed no difference in the two CG evaluation moments (0.807; n=32) (Table 3), confirming the efficacy of music therapy and excluding the possibilities of other sources of influence. There was a similar result in a music therapy study with active methods, conducted with Graduation and Post-Graduation students in a Brazilian Public University, showing stress level reduction, evaluated by LSSI pre/post intervention (Panacione, 2012).

Another study that checked the effects of an active music therapy program, applied in a group of women working in different areas of a Brazilian private hospital, showed a statistically meaningful decrease (variation = - 60%, *p*<0.001) in stress levels (Taets *et al*., 2013). In spite of the methodological differences regarding the format of interventions and varied contexts, the results of the present study match results found by the authors mentioned above.

In Music in Medicine, the receptive use of music suggests potential therapeutic effects. These effects are attributed to the capacity of the music to reduce stress and modulate levels of excitement. A study conducted with volunteer women evaluated the effect of music listening with earphones on their stress level. It showed that listening to relaxing music before a stressful experience affects domains (endocrine, autonomous, cognitive, and emotional systems) differently. Listening to music before a patterned stressor affected the autonomic nervous system, causing a faster recovery and, in smaller scale, affected the endocrinal response and psychological stress. The beneficial effect of music in the hypothalamus- pituitary- adrenal axis (HPA) depends on the situational context and chronology of the events (*versus* before, during or after stress). Despite the benefits of the use of music on the human body, the results of the aforementioned study don´t support the notion that music should be used as a tool for stress management in a context of stressor anticipation (Thoma *et al*., 2013). It is important to note that the methodology applied in the study conducted by Thoma *et al*. (2013) required a passive attitude from the participants, with earphones, without the possibility of self-expression during the listening. This approach is methodologically different from the one used in the present study, in which the patients had active participation in the process of music creation. This may justify the differences between the results found in the present study and the ones conducted by the aforementioned authors.

Other two studies evaluated the effects of live music, without the use of earphones, during 20 minutes, in women submitted to embryo transfer. The results showed positive effects on acute stress (Murphy *et al*., 2014) and anxiety levels (Moragianni *et al*., 2009). Even if one considers the differences between the approaches and methodology of these studies and the present one, the results reinforce our findings. Stress and anxiety are associated events (Vignola, 2013; Vignola & Tucci, 2014), and they are expressively correlated with infertility (Peterson *etal*., 2014; Gusmão *et al*., 2020). Murphy *et al*. (2014) showed that the harp therapy, applied in the form of listening to music, decreased the state of anxiety of the participants of the study. Contrarily, other studies reported that the use of music had no meaningful impact on the decrease of anxiety levels of women submitted to IVF (Stocker *et al*., 2016; Aba *et al*., 2017).

The participants’ level of anxiety was not evaluated in the present study. However, considering the close relation between stress and anxiety, it is worth to point the methodological differences concerning the application of music between the studies of Stocker *et al*. (2016) and Aba *et al*. (2017), and the study of Moragianni *et al*. (2009) and Murphy *et al*. (2014), carried out with women undergoing high complexity procedures for infertility treatment.

In the studies carried out by Stocker *et al*. (2016) and Aba *et al*. (2017), the listening occurred from recorded songs and the use of earphones. Differently, Moragianni *et al*. (2009) and Murphy *et al*. (2014) used “live music”, that is, the songs were played and presented to the participants during the moment of the embryo transfer without the use of earphones. Even if there are similarities in the categorization of the referred studies, that is, they all are included in the Music in Medicine approach (Bradt *et al*., 2013), the use of earphones or the lack of them probably led to controversial results regarding anxiety reduction.

It is important to consider that women undergoing IVF/ICSI not only feel the collateral effects of medications (Finotti, 2011), but also have their bodies expressively invaded due to more aggressive, relatively painful and stressful medical evaluation mechanisms (Moura-Ramos, 2011). It is believed that the use of earphones as presented in the studies of Stocker *et al*. (2016) and Aba *et al*. (2017) may have been perceived (unconsciously or not) as an invasive element due to the emotional fragility of the patients during this process. Consequently, the resulting data have no statistical meaning.

On the other hand, considering the differences between the approaches employed in the studies of Moragianni *et al*. (2009) and Murphy *et al*. (2014) and the one applied in the present study, the 3 studies have in common the “live” presence of music, that is, CDs or MP3 were not used as elements of mediation in the intervention process. This probably led to similar results. However, similar studies, in both approaches, would contribute for a better evaluation of the results.

Another important aspect is the fact that the potential therapeutic effects of listening to music are linked to fac- tors of personality of the subjects and to the individual dif- ferences that show a variety of music preferences (Chanda & Levitin, 2013) serving, therefore, as mediators of its effects. Music has an absolutely personal and non-transferable meaning associated to a specific time (Blasco,1999). That is, what is relevant (in hearing) for a subject in a specific moment, may not have the same meaning in a posterior moment, which is explained by the polysemy present in music (Barcellos & Santos, 1996).

Music is experienced differently by each individual, according to their musical taste, familiarity with style, genre, and other aspects associated to the structure of the composition (Fancourt *et al*., 2014; Fleury *et al*., 2016). Therefore, the effects that music may cause on the subject have a wide spectrum. It is necessary strictness to identify which of the elements (harmony, rhythm, timbre, texture, among others) are responsible for biological and psychological changes. There is a wide range of types of musical intervention. However, there is a predominant tendency in investigations that involve music to categorize the interventions exclusively as “recorded music” or “music creation”, as if the activity could be understood as an only entity (Fancourt *et al*., 2014). The lack of further details of the music elements present in the compositions used in investigations is a factor that hinders the replication of the methodology in other samples, making it more difficult for a better comprehension of the music effects (Fancourt *et al*., 2014; Fleury *et al*., 2016).

It is also relevant to observe that Fancourt *et al*. (2014), alert about the existence of a series of scientific papers that categorize the interventions with music incorrectly. They cite the works of Conrad *et al*. (2007), Peng *et al*. (2009), and White (1992), among others, who used the term “music therapy” equivocally. The same may be seen in the studies from Aba *et al*. (2017), and Stocker *et al*. (2016). These questions could easily be solved with the presence of a music therapist in the research team, since this professional is qualified to practice in the hybrid field of music and therapy (Bruscia, 2000; Bradt *et al*., 2013). This is another fact that makes it difficult for a better understanding of the therapeutic effects of music and music therapy on human health.

A randomized clinical trial conducted by Vianna *et al*. (2011), with the participation of 94 mothers of premature newborns, showed the positive impact of music therapy in the increase of breastfeeding index, with statistically significant differences between the Music Therapy Group and the Control Group in the first follow-up appointment (*p*=0.03). The authors concluded that active music therapy can be useful to increase the breastfeeding index of mothers of premature newborns (Vianna *et al*., 2011). Although the studies of Vianna *et al*. (2011) don’t discuss the psychological suffering of these mothers, it is known that the experience of giving birth to a premature child is a stressful situation for the woman. Stress is also one of the factors that negatively influences breastfeeding (Rodrigues *et al.*, 2013). It may occur by interference in the production of breast milk, once the stress hormones are capable of inhibiting the action of prolactin or oxytocin (Levy & Bértolo, 2012; Nilsson, 2009). They can also act as an inhibitor of the mother´s ability to cope with breastfeeding itself. In this perspective, there is the hypothesis that the music therapy interventions probably acted in stress reduction at some level, consequently raising the breastfeeding index. However, studies referring to this association - stress, breastfeeding, (inter)active music therapy - must be carried out to acquire evidence to prove this hypothesis.

The assisted music composition (Barcellos, 2015), main technique applied in this study, gave conditions for the women in IG to express themselves verbally and musically, and to listen to themselves, regarding their fears, doubts, and concerns referring both to the infertility and to the possible result of the ART, enhancing the emotional support the women needed. The results obtained are relevant because they can open new possibilities of contribution for women undergoing ART. They can also fill a blank in the Brazilian literature concerning the application of music therapy with this population, this being a pioneer study in this medical specialty.

Furthermore, the present study offers contributions to fields that are correlated with Music Therapy, such as Medicine, Nursing, and Psychology, clarifying the differences related to the use of Music in Medicine and Music Therapy in Medicine, the theoretical-methodological concepts, and bringing more elucidation for new researches that use music. It is also relevant to emphasize the emergent need for a mental health professional to monitor their emotional state and to support them.

## CONCLUSIONS

The results obtained in this study allowed concluding that interactive music therapy was effective in the reduction of the stress level of IG when compared to CG, at the end of the study. The interactive approach and the use of techniques from Musical Exploration, Non-referential Instrumental Improvisation and Assisted Music Composition were shown to be efficient in this study. However, more investigation should be done in order to guarantee data generalization. The limitations of the study, as the fact that it was conducted in a single medical center specialized in assisted reproduction, make the generalization difficult. Multicenter studies with the involvement of a wider population may provide more information.

## Footnotes

[1] The author, Eliamar Aparecida de Barros Fleury e Ferreira, also identifies herself as Fleury in her publications.
